# Control of Raw Pork Liver Sausage Production Can Reduce the Prevalence of HEV Infection

**DOI:** 10.3390/pathogens10020107

**Published:** 2021-01-22

**Authors:** Paolo Ripellino, Enea Pianezzi, Gladys Martinetti, Cinzia Zehnder, Barbara Mathis, Petra Giannini, Nicola Forrer, Giorgio Merlani, Harry R. Dalton, Orlando Petrini, Florian Bihl, Stefano Fontana, Claudio Gobbi

**Affiliations:** 1 Neurocenter of Southern Switzerland, 6900 Lugano, Switzerland; claudio.gobbi@eoc.ch; 2 Laboratory of Microbiology, Ente Ospedaliero Cantonale, 6500 Bellinzona, Switzerland; enea.pianezzi@eoc.ch (E.P.); gladys.martinettilucchini@eoc.ch (G.M.); 3 Synlab Ticino, 6934 Bioggio, Switzerland; cinzia.zehnder@synlab.com; 4 Unilabs Ticino, 6932 Breganzona, Switzerland; barbara.mathis@unilabs.com; 5 Cantonal Food Control Authority, 6500 Bellinzona, Switzerland; petra.giannini@ti.ch (P.G.); nicola.forrer@ti.ch (N.F.); 6 Cantonal Service for Health, 6500 Bellinzona, Switzerland; giorgio.merlani@ti.ch; 7 University of Applied Sciences and Arts of Southern Switzerland, 6501 Bellinzona, Switzerland; hardalton@gmail.com; 8 Royal Cornwall Hospital, Truro TR1 3LJ, UK; orlando@poleconsult.com; 9 San Giovanni Hospital, 6500 Bellinzona, Switzerland; florian.bihl@eoc.ch; 10 Hôpitaux Universitaires de Genève, 1205 Genève, Switzerland; 11 Blood Transfusion Service CRS Southern Switzerland, 6900 Lugano, Switzerland; stefano.fontana@itransfusion.ch; 12 Faculty of Biomedical Sciences, Università della Svizzera Italiana (USI), 6900 Lugano, Switzerland

**Keywords:** hepatitis E, epidemiology, food chain control, public health, one health, sausage, raw liver

## Abstract

After an acute hepatitis E (HEV) outbreak in Southern Switzerland, in January 2017 the local public health authorities started an active program of food chain control and public education. In this retrospective study, we analysed all laboratory-confirmed acute cases of HEV infection diagnosed between 2014 and 2020. In the period before the public health intervention, the number of cases increased steadily from 2014 (4 of 40 tests, 10%) reaching a peak in the last quarter of 2016 (42 of 285 tests, 14.7 %). Afterwards, the number of positive cases decreased steadily, reaching its lowest value (0.3%) in the second quarter of 2019. There was a statistically significant difference between the frequency of positive cases and period of testing, i.e., before and after the introduction of the public health interventions. Our study shows that active public health measures to control sausages containing raw pork liver can reduce the prevalence of HEV infection.

## 1. Introduction

Hepatitis E (HEV) is the most prevalent cause of acute hepatitis in the world [1]. In developed countries, HEV infection occurs mainly through zoonotic transmission or, more rarely, contaminated blood products [1]. Pigs are the main asymptomatic reservoir [1], with a variable seroprevalence [2]. In Switzerland, the estimated overall seroprevalence in pigs is approximately 60% [3,4]; in humans (healthy blood donors) it is 20%, but higher values have been reported from Southern Switzerland (>30%), where the prevalence may reach almost 60% in some districts [5]. 

Numerous studies have shown that meat products from domestic pigs and wild boars, especially pork sausages containing raw liver, are contaminated with HEV [2,6,7,8,9,10].

**Figure 1:**
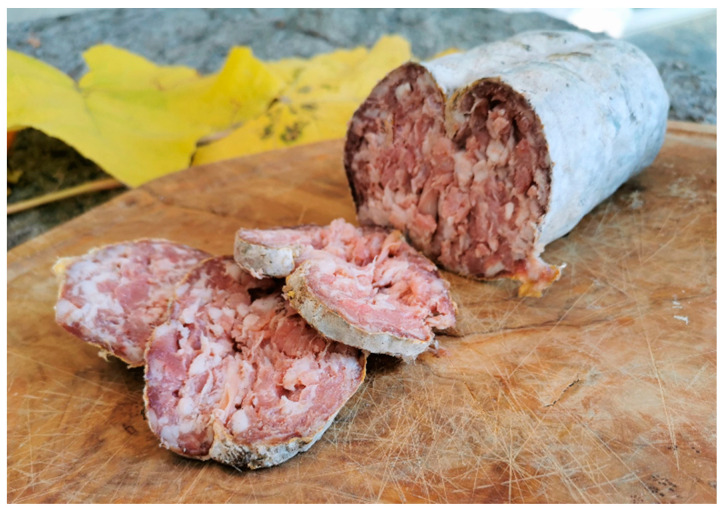
“Mortadella di fegato crudo”, a historical, traditional local product of Southern Switzerland containing raw pork liver. The production rules and consumption recommendations for this sausage were changed at the beginning of 2017.

The percentage of HEV-contaminated food products of porcine origin can vary from less than 1% to more than 50%, depending on the country and the local products. It has also been suggested that regional variations in the human seroprevalence may depend on the consumption of raw traditional dry-cured pork products, especially those containing raw pork liver (Figure 1) [6,7,11]. For example, in France HEV-RNA was present in up to 30% of food containing raw pork liver, such as sausages [7]. A screening program conducted in Switzerland in 2016 [8] detected HEV-RNA in 11% of all ready-to-eat meat products, mainly liver sausages (19%). 

In Southern Switzerland, raw sausages sampled at retail level (Figure 1) were screened for the presence of HEV by quantitative real-time polymerase chain reaction (RT-PCR) [9]. HEV was detected in 12 (11.8%) of 102 sausage products containing raw liver, but not in any other pork sausages without liver. Probably a low percentage of infected liver is sufficient to contaminate an entire batch [12]. To date, the only efficient control option for HEV infection from consumption of HEV contaminated meat products is sufficient heat treatment, as HEV can be inactivated by cooking the meat at 71 °C for 20 min [13]. 

A previous observational study carried out in Southern Switzerland [14] identified 141 acute cases of HEV, of whom approximately 30% complained of neurological symptoms. Almost all these patients reported consumption of raw pork meat products in the month preceding the appearance of symptoms. In one case an HEV strain isolated from the patient’s stool sample was identical with the one detected in a sausage that he had eaten [11]. These observations and the high number of cases of acute HEV (the majority requiring hospitalization) identified in our region led the local Public Health Authority to introduce a “One-Health” action plan [15,16] to reduce the amount of HEV contaminating the food chain and to educate meat consumers. This started in January 2017 and was focused on the reduction of HEV-contaminated food on the market and appropriate consumer information (Table 1). 

In the first quarter of 2017, the local Food Control Authority provided recommendations to meat producers to include HEV in the Hazard Analysis and Critical Control Point (HACCP) system for products containing raw pork liver. Since April 2017 meat producers were required to avoid the inclusion of raw pig liver as an ingredient in cured sausages, to substitute it with the liver of other animals (e.g., calf), or to check for the presence of HEV in products with raw pork liver at the time of batch release. In addition, they had to inform consumers about the need to adequately cook products containing raw pork liver. The consumer information campaign was primarily addressing vulnerable groups of the population (“YOPI”, young, old, pregnant, and immunosuppressed). These were invited to thoroughly cook meat and offal, especially pork, wild boar and deer meat products. In May 2017, the Cantonal Doctor informed all medical doctors active in Southern Switzerland on the modality of HEV transmission and its possible hepatic and extra-hepatic clinical manifestations.

The aim of this retrospective epidemiological study was to determine the effect of the introduction of a public health intervention on the number of HEV cases in humans in Southern Switzerland.

## 2. Results

Within the study period, 6068 subjects were tested for acute HEV by serology (IgM antibodies) and RT-PCR. Four cases had increased liver enzymes and reactive serum HEV IgM+ and IgG+, but were considered as negative because they had a concomitant Cytomegalovirus infection, a possible cause of false positivity [17]. 

The characteristics of the acute HEV subjects and the results of serology and PCR tests are summarised in Table 2. 

**Figure 2:**
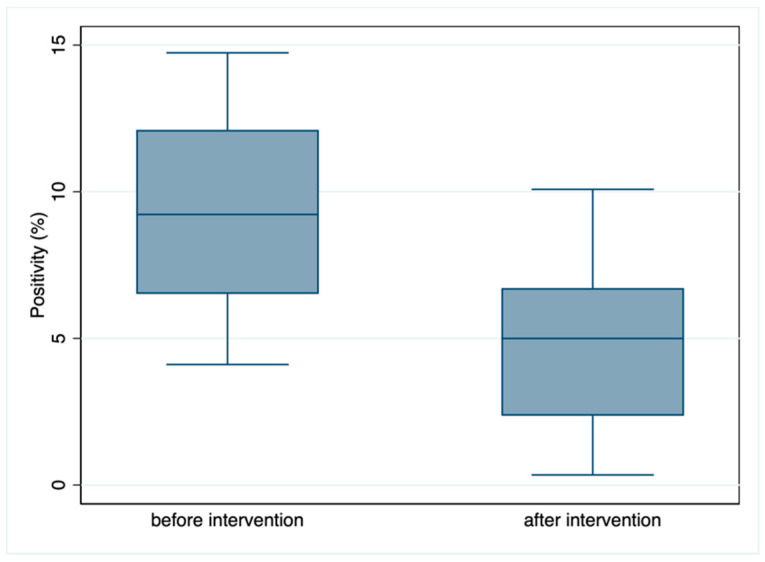
Boxplot of the serological IgM positivity (frequency of acute cases) observed before and after the introduction of the public health intervention. Blue line: median; box: interquartile range; whiskers: 95% confidence interval of the median. The y-axis indicates the percentage of positive cases.

Overall, 375/6068 (6.1%) subjects had a serologically confirmed acute HEV infection (Table 2; Figure 2). Of these 375 acute cases, 55 (14.6%) had an HEV infection confirmed by RT-PCR (Table 2, Figure 2). Four of the 55 viraemic patients were initially HEV IgM negative, but seroconverted (IgM+ and IgG+) within 2 weeks. Genotyping was performed in 34 (61%) of the viraemic cases and was successful in 18 viraemic samples. Only HEV genotype 3 was detected.

A test for independence indicated a statistically significant negative association between the number of serologically confirmed acute positive cases and period of testing (9.9% positive tests before vs. 5% after the intervention; Pearson chi2 = 44,875, *p* = 0.0000; see Figure 2).

**Figure 3:**
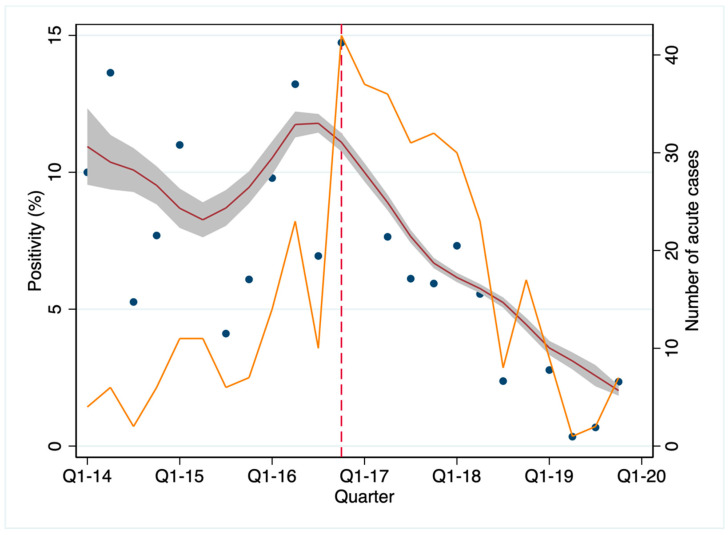
Acute HEV cases (% of tests) occurring in Southern Switzerland in the study period, by quarters of years. The red line was computed using a local polynomial smoothing, weighted by the total number of tests; this line represents the fluctuation over time in the percentage of positive cases. Grey shading: 95% confidence interval; blue dots: observed positivity rates at each measurement time; orange line: number of acute cases by quarter of year. The vertical dashed line indicates the start of the public health intervention in January 2017.

The positivity (percentage of acute HEV cases) for each quarter of year is shown in Figure 3. 

The number of acute HEV cases increased from the first quarter 2014 and the positivity reached its peak (42/285, 14.7%) in the fourth quarter of 2016. With the start of the active program of food chain control, the number of cases decreased, and in the whole of 2019, only 19/1206 tested cases (1.5%) were positive. 

Three patients died for complications directly caused by HEV. Two were immunocompetent patients with pre-existing Child C cirrhosis. They developed acute or chronic damage and the classical complications of end stage liver failure (hepatic encephalopathy, acute kidney injury, bacterial peritonitis). The third case was an immunosuppressed transplanted patient who developed chronic HEV infection. Despite continuous ribavirin treatment and reduction in the dose of immunosuppressants, the patient never cleared HEV and died three years later.

## 3. Discussion

This is the first study investigating the impact of a public health intervention in an industrialized country, undertaken during a hepatitis E outbreak and aiming at reducing the number of cases. 

The data suggest that active intervention in the food chain in combination with a public information campaign may help reduce the number of HEV infections in the local population. We observed an increase in symptomatic HEV cases from 2014 until the end of 2016, but from the start of the active program of food chain control (January 2017) the number of positive cases progressively declined (Figure 3). 

Southern Switzerland is a favourable setting to perform an epidemiological study such as this. The region has well defined geographic limits, being delineated by the Alps on the North and the Italian borders on the West, South and East borders; it has a rather stable population, and a distinct culinary culture, in which the consumption of raw pork sausages is comparatively high. In addition, during the study period, only three microbiology laboratories performed HEV tests using identical methods, and clinicians were aware of HEV as a potential diagnosis in patients presenting with hepatitis and/or acute neurological injury. Taken together, these factors facilitated consistent and robust data capture.

The pro-active attitude of the local Cantonal Public Health Department, which sets guidelines in food production processes and conducted an active information campaign, was pivotal in reducing the number of cases. Soon after the public health intervention, the number of newly diagnosed cases of HEV started to decrease (Figure 3). The decrease in the number of cases, however, is unlikely to be due to a testing bias. In fact, after the public health intervention, the number of HEV tests increased continuously (up to 180/month at the end of 2017), while the rate of positive cases decreased steadily from 14.7% at the end of 2016 to 0.3% in mid-2019 (Figure 3). These data suggest a true reduction in circulating HEV in the human population following the intervention.

Recent data suggest that the morbidity of HEV infection is higher than previously thought, with a mortality approaching 4% in diagnosed cases [18]. In our cohort, however, only 3 of 375 cases (0.8%) died because of complications directly related to hepatitis E.

The European Food Safety Authority (EFSA) published a scientific opinion on HEV and the possibilities of its transmission [19], highlighting the need to improve HEV detection methods, including infectivity assays and consensus molecular typing protocols. Currently, the European Association for the Study of the Liver (EASL) recommends using a combination of serology and nucleic acid testing to diagnose acute HEV infection [20]. There is still uncertainty regarding the type of antibody assay that should be used, while for nucleic acid amplification (NAT)-based assays a consensus has been reached [21]. 

HEV is not a notifiable disease in many European countries, but in recent years, cases of zoonotic HEV have been increasingly reported in Europe [2,22]. Based on the number of symptomatic human HEV cases observed in Switzerland in the last few years [9,14,23], in January 2018 Switzerland introduced mandatory notification for HEV, and since November 2018 all blood products are screened for HEV by PCR in pools of 24 (or less) blood donations. 

More research is required on HEV epidemiology and control in pig herds to minimise the proportion of pigs that remain viraemic or carry high viral loads in their intestine at the time of slaughter. A Swiss study, aiming at determining the risk of foodborne transmission by analysing retail meat products [10], concluded that successful HEV human infection may occur by ingestion, as 1% of all servings investigated contained high HEV loads. The authors estimated the number of cases in the whole Switzerland in one year to be 1500, considering that acute illness develops in approximately 5% of susceptible consumers. In Southern Switzerland, the annual burden of hepatitis E, estimated in terms of Disability Adjusted Life Years (DALY), increased from <5 DALY per 100,000 inhabitants before 2012 to >50 DALY per 100,000 inhabitants in 2015 [10].

Our study has some limitations. The retrospective nature of the study is prone to bias. For instance, we identified only symptomatic cases that were tested; thus, we may have missed additional symptomatic cases. In addition, many cases of HEV infection may occur asymptomatically, thus being undetected. Our observations may have underestimated the efficacy of the intervention, and the true amount of circulating HEV in humans may be higher. The second main limitation is the lack of a standardized method for serology. The different sensitivity and specificity of the available kits [24] may yield different results even in the same population. For example, the seroprevalence in Swiss blood donors varied from 4.9% to 21% depending on the kit used [25]. A large meta-analysis [24] concluded that the observed heterogeneity in seroprevalence rates in Europe is mainly a consequence of the assay employed. With the choice of the testing assay, we may thus have over- or underestimated the true positivity rates. On the other hand, the consistent use of the same serology and PCR tests during the whole study period in all laboratories may have, at least to some extent, reduced this risk of bias. 

In summary, our study provides evidence that a public health intervention aimed at educating the population and modifying the production of the traditional sausages made with raw pork liver effectively reduced the number of human HEV cases in Southern Switzerland. Further research, based on international collaborations (such as the “HEV-net” network [26]) and multimodal campaigns (such as the one recently started in Portugal [27]), is required to confirm our findings in other geographical locations and in a broader setting.

## 4. Materials and Methods

We retrospectively analysed data obtained from three clinical Microbiology laboratories that serve public and private hospitals, family physicians, and private health institutions and cover the whole geographical area of Southern Switzerland (350,000 inhabitants as of 2020). We included subjects tested for acute hepatitis E by IgM serology and/or real-time quantitative HEV RNA reverse transcriptase PCR (RT-PCR) between 1 January 2014 and 31 December 2019. Subjects were considered true positive only if they had increased liver enzymes and reactive serum anti-HEV IgM and anti-HEV IgG, and/or HEV RNA detected in serum by RT-PCR. Subjects with low HEV IgM positivity not confirmed by a repeated serology test after 1 month, or subjects with reactive serum anti-HEV IgM and anti-HEV IgG but suffering from another concomitant infection, were considered negative.

### 4.1. Laboratory Analysis

Sera from patients with suspected HEV infection were tested for HEV IgG and IgM using Dia.Pro kits (Diagnostic Bioprobes srl, Italy). The sensitivity of this kit is 98% for IgG and 72% for IgM, and its specificity is 96% for IgG and 100% for IgM [28]. Real-time quantitative PCR and HEV genotype determination were performed as previously described by Fraga et al. [23]. 

### 4.2. Statistical Analysis

A Chi-square test for independence was used to investigate any association between the number of positive cases and period of testing. Boxplots were used to display positivity rates, and a local polynomial regression smoothing, weighted according to the number of positive cases, was used to display the time course of the positivity. All statistical tests were carried out using Stata ver. 16 (StataCorp, College Station, TX, USA).

## Figures and Tables

**Table 1:** Public intervention to control food chain supply and inform the population.

Date	Intervention	Expected Aim
November 2016	Meeting of representatives of the Cantonal Health Service, the Cantonal Veterinary Office, and the Cantonal Food Control Agency	Develop a One Health action plan to reduce human HEV exposure in the food chain at Cantonal (sub-regional) level
December 2016	Involvement of the Federal Food Safety and Veterinary Office (FSVO)	Establish effective measures to protect consumers’ health and agree on appropriate risk communication strategies for local authorities
January–April 2017	Start of the active program with the involvement of the Swiss Meat Associations	Include HEV as a hazard in the HACCP * system and provide effective control measures
April 2017	Press release with recommendations by the local authorities to: (a) all meat producers: detailed description of the mandatory changes in the production of sausages containing raw pork liver (b) all medical doctors active in Southern Switzerland: explanation of the modality of HEV infection and its possible hepatic and extra-hepatic clinical manifestations	Inform the local population in order to prevent HEV infections. In particularly, protect YOPI ** people
1 May 2017	Publication of an information letter from FSVO to inform the population and the local authorities of other regions of Switzerland on the risk of HEV infection after consumption of “high risk products”	Inform the population and the local authorities of other regions of Switzerland
May 2018–May 2021	Research project: HEV along the human food chain: investigations into spread, genetic diversity and molecular tracing.	Achieve a better HEV risk assessment.

* HACCP = Hazard Analysis and Critical Control Point system; ** YOPI = young, old, pregnant, and immunosuppressed.

**Table 2:** Age and gender of acute hepatitis E (HEV) subjects, and positivity rates of serology and PCR tests.

	Before Intervention	After Intervention
Age (years; median (quartiles))	58 (45–70)	55 (44–66)
**Gender (%)**		
Female Male	38.1 61.9	46.6 53.4
**Serology tests**		
Positive (N (total no. of tests))	142 (1434)	233 (4634)
Overall positivity (%)	9.9	5
**PCR tests**	184	75
Positive (N (total no. of tests))	32 (184)	23 (75)
Overall positivity (%)	17.4	30.7

95% CI: 95% confidence interval of the mean.

## Data Availability

The data presented in this study are available on request from the corresponding author.

## References

[1] Kamar N., Izopet J., Pavio N., Aggarwal R., Labrique A., Wedemeyer H., Dalton H.R. (2017). Hepatitis E virus infection. Nat. Rev. Dis. Primers.

[2] Salines M., Andraud M., Rose N. (2017). From the epidemiology of hepatitis E virus (HEV) within the swine reservoir to public health risk mitigation strategies: A comprehensive review. Vet. Res..

[3] Wacheck S., Sarno E., Martlbauer E., Zweifel C., Stephan R. (2012). Seroprevalence of anti-hepatitis E virus and anti-Salmonella antibodies in pigs at slaughter in Switzerland. J. Food Prot..

[4] Burri C., Vial F., Ryser-Degiorgis M.P., Schwermer H., Darling K., Reist M., Wu N., Beerli O., Schoning J., Cavassini M. (2014). Seroprevalence of hepatitis E virus in domestic pigs and wild boars in Switzerland. Zoonoses Public Health.

[5] Niederhauser C., Widmer N., Hotz M., Tinguely C., Fontana S., Allemann G., Borri M., Infanti L., Sarraj A., Sigle J. (2018). Current hepatitis E virus seroprevalence in Swiss blood donors and apparent decline from 1997 to 2016. Euro Surveill..

[6] Berto A., Martelli F., Grierson S., Banks M. (2012). Hepatitis E virus in pork food chain, United Kingdom, 2009–2010. Emerg. Infect. Dis..

[7] Pavio N., Merbah T., Thebault A. (2014). Frequent hepatitis E virus contamination in food containing raw pork liver, France. Emerg. Infect. Dis..

[8] Moor D., Liniger M., Baumgartner A., Felleisen R. (2018). Screening of Ready-to-Eat Meat Products for Hepatitis E Virus in Switzerland. Food Environ. Virol..

[9] Giannini P., Jermini M., Leggeri L., Nuesch-Inderbinen M., Stephan R. (2018). Detection of Hepatitis E Virus RNA in Raw Cured Sausages and Raw Cured Sausages Containing Pig Liver at Retail Stores in Switzerland. J. Food Prot..

[10] Muller A., Collineau L., Stephan R., Muller A., Stark K.D.C. (2017). Assessment of the risk of foodborne transmission and burden of hepatitis E in Switzerland. Int. J. Food Microbiol..

[11] Kubacki J., Fraefel C., Jermini M., Giannini P., Martinetti G., Ripellino P., Bernasconi E., Sidler X., Stephan R., Bachofen C. (2017). Complete Genome Sequences of Two Swiss Hepatitis E Virus Isolates from Human Stool and Raw Pork Sausage. Genome Announc..

[12] Rose N., Lunazzi A., Dorenlor V., Merbah T., Eono F., Eloit M., Madec F., Pavio N. (2011). High prevalence of Hepatitis E virus in French domestic pigs. Comp. Immunol. Microbiol. Infect. Dis..

[13] Barnaud E., Rogee S., Garry P., Rose N., Pavio N. (2012). Thermal inactivation of infectious hepatitis E virus in experimentally contaminated food. Appl. Environ. Microbiol..

[14] Ripellino P., Pasi E., Melli G., Staedler C., Fraga M., Moradpour D., Sahli R., Aubert V., Martinetti G., Bihl F. (2020). Neurologic complications of acute hepatitis E virus infection. Neurol. Neuroimmunol. Neuroinflamm..

[15] Destoumieux-Garzon D., Mavingui P., Boetsch G., Boissier J., Darriet F., Duboz P., Fritsch C., Giraudoux P., Le Roux F., Morand S. (2018). The One Health Concept: 10 Years Old and a Long Road Ahead. Front. Vet. Sci..

[16] Mrzljak A., Dinjar-Kujundzic P., Jemersic L., Prpic J., Barbic L., Savic V., Stevanovic V., Vilibic-Cavlek T. (2019). Epidemiology of hepatitis E in South-East Europe in the “One Health” concept. World J. Gastroenterol..

[17] Fogeda M., de Ory F., Avellon A., Echevarria J.M. (2009). Differential diagnosis of hepatitis E virus, cytomegalovirus and Epstein-Barr virus infection in patients with suspected hepatitis E. J. Clin. Virol..

[18] Wallace S.J., Swann R., Donnelly M., Kemp L., Guaci J., Murray A., Spoor J., Lin N., Miller M., Dalton H.R. (2020). Mortality and morbidity of locally acquired hepatitis E in the national Scottish cohort: A multicentre retrospective study. Aliment. Pharmacol. Ther..

[19] Hazards E.P.o.B., Ricci A., Allende A., Bolton D., Chemaly M., Davies R., Fernandez Escamez P.S., Herman L., Koutsoumanis K., Lindqvist R. (2017). Public health risks associated with hepatitis E virus (HEV) as a food-borne pathogen. EFSA J..

[20] European Association for the Study of the Liver (2018). EASL Clinical Practice Guidelines on hepatitis E virus infection. J. Hepatol..

[21] Baylis S.A., Blumel J., Mizusawa S., Matsubayashi K., Sakata H., Okada Y., Nubling C.M., Hanschmann K.M., HEV Collaborative Study Group (2013). World Health Organization International Standard to harmonize assays for detection of hepatitis E virus RNA. Emerg. Infect. Dis..

[22] Aspinall E.J., Couturier E., Faber M., Said B., Ijaz S., Tavoschi L., Takkinen J., Adlhoch C., The Country Experts (2017). Hepatitis E virus infection in Europe: Surveillance and descriptive epidemiology of confirmed cases, 2005 to 2015. Euro Surveill..

[23] Fraga M., Doerig C., Moulin H., Bihl F., Brunner F., Mullhaupt B., Ripellino P., Semela D., Stickel F., Terziroli Beretta-Piccoli B. (2018). Hepatitis E virus as a cause of acute hepatitis acquired in Switzerland. Liver Int..

[24] Hartl J., Otto B., Madden R.G., Webb G., Woolson K.L., Kriston L., Vettorazzi E., Lohse A.W., Dalton H.R., Pischke S. (2016). Hepatitis E Seroprevalence in Europe: A Meta-Analysis. Viruses.

[25] Schnegg A., Burgisser P., Andre C., Kenfak-Foguena A., Canellini G., Moradpour D., Abravanel F., Izopet J., Cavassini M., Darling K.E. (2013). An analysis of the benefit of using HEV genotype 3 antigens in detecting anti-HEV IgG in a European population. PLoS ONE.

[26] Mulder A.C., Kroneman A., Franz E., Vennema H., Tulen A.D., Takkinen J., Hofhuis A., Adlhoch C., Members of HEVnet (2019). HEVnet: A One Health, collaborative, interdisciplinary network and sequence data repository for enhanced hepatitis E virus molecular typing, characterisation and epidemiological investigations. Euro Surveill..

[27] Mesquita J.R., Myrmel M., Stene-Johansen K., Overbo J., Nascimento M.S. (2016). A Public Health initiative on hepatitis E virus epidemiology, safety and control in Portuga—Study protocol. BMC Infect. Dis..

[28] Norder H., Karlsson M., Mellgren A., Konar J., Sandberg E., Lasson A., Castedal M., Magnius L., Lagging M. (2016). Diagnostic Performance of Five Assays for Anti-Hepatitis E Virus IgG and IgM in a Large Cohort Study. J. Clin. Microbiol..

